# Sustained Viremic Control in HIV-Infected Patient: Case Report from Nepal

**DOI:** 10.1155/2018/5279595

**Published:** 2018-03-13

**Authors:** Sundar Khadka, Subhash Dhital, Roshan Pandit, Anup Bastola

**Affiliations:** ^1^HIV Reference Unit, National Public Health Laboratory, Kathmandu, Nepal; ^2^Sukraraj Tropical & Infectious Disease Hospital, Teku, Kathmandu, Nepal

## Abstract

A case of human immunodeficiency virus (HIV) infection is described from Nepal with constant maintenance of CD4 count and HIV-RNA level below the lower detection threshold for more than ten years. The case was diagnosed of HIV positive in the year 2008. He had his viral load estimation performed every year since then which was always below lower detection limit and remained healthy without treatment. The patient also had not any kinds of opportunistic infection till date. He is married now and has not transmitted the disease to his wife.

## 1. General Background

The incidence of HIV infection is increasing globally. WHO reported 34 million people living with HIV at the end of 2011 [1]. The prevalence of HIV infection among adults was reported to be 0.20% in the year 2014 in Nepal [2]. It is estimated that 7,400 new cases of HIV are added each day worldwide. In the year 2011, 1.7 million deaths occurred due to HIV-AIDS, and in the same year, 2.5 million new cases were diagnosed of HIV-AIDS [3].

Many attempts have been made to control as well as provide successful treatment to the deadly disease, yet there is not an easy-to-follow strict regimen for treatment. One of the major causes of the situation the unclear understanding about the various complicated modes of HIV pathogenesis. Different research works suggest that the previously known and newly emerging HIV pathogenesis is somehow different due to which some people can constantly control over viral replication thus resulting in long-term nonprogressors and elite controllers [4].

Elite controllers are those HIV-positive individuals that show plasma HIV-RNA value persistently below 50 copies/ml or below the lower detection threshold of clinical assays without antiretroviral therapy throughout the course of infection or for at least 12 months [4–7]. On the other hand, long-term nonprogressors are those individuals showing low-detectable plasma viremia, that is, less than 5000 HIV-RNA copies/ml [4].

Here, we presented a case that has an extraordinary control over HIV replication with constant maintenance of CD4 count and HIV-RNA level below the lower detection threshold for more than 10 years. The case was identified in 2016 by retrospective analysis of the laboratory report and clinical history explained by the patient. Though this type of case has been reported from developed countries, to our knowledge, this is the first case report from a developing country like Nepal.

This type of case is important because this may aid in understanding the pathogenesis of HIV in elite controllers that is still under investigation. Furthermore, these candidates may be used for therapeutics and vaccine development which may contribute to the establishment of definite treatment guidelines for HIV-related cases.

## 2. Case Description

A 40-year-old male resident from Kathmandu, Nepal, was tested positive for HIV infection by the ELISA method in 2008. In addition to ELISA, supplementary testing for HIV serology was done by using rapid test kits (Determine, Uni-Gold, and Stat-Pak) which are recommended by national as well as WHO guidelines for confirmation. In the described case, all the three tests were positive; hence, the case was considered as HIV infected and requested for follow-up clinic visit for CD4 testing, viral load, and opportunistic infections before initiation of antiretroviral therapy.

He shared that he was engaged in needle sharing while injecting drugs, possibly a mode of transmission. In the initial finding, CD4 count was 450 cells/*µ*l, and the viral load was less than the detectable limit. He had his CD4 count performed every year since 2008 and viral load testing, which was always below lower detection threshold. The detailed data is presented in Table 1. In 2016, western blot investigation was performed after finding of strikingly continuous undetectable viral load and high CD4 count to reassess the HIV status. The result of HIV western blot was positive. Until this time, the patient was not enrolled on ART. The patient had not any kinds of opportunistic infection till date and had not suffered from any kinds of serious illness. He is married now, and his partner is HIV negative till now.

All the tests were performed at National Public Health Laboratory (NPHL), Teku, Kathmandu. CD4 count was performed using BD fluorescent-activated cell sorter system (BD Biosciences, San Jose, CA, USA). For this, whole blood sample was collected in EDTA vial, and the count was performed as per the manufacturer's instructions. For viral load testing, whole blood sample was collected, and plasma was used for further testing. Viral RNA was extracted using QIAamp Viral RNA Mini Kit or Roche's High Pure Viral Nucleic Acid Kit. The extracted RNA was then analyzed by real time polymerase chain reaction (RT-PCR) using Corbett Rotor-Gene 6000, COBAS®TaqMan® 48 Analyzer.

## 3. Discussion

Elite controllers are very rare group of populations harboring human immunodeficiency virus. It is known that such type of population possess HIV virus but could not transmit infection to other individuals. The mechanism by which they could control viral replication has not been fully understood till date [4, 8, 9]. Some studies suggest that the host immune factor plays an important role in controlling replication while others suggest that it is the result of infection with defective viral strain [10]. Finding such type of cases may aid in understanding the mechanism involved in the control of HIV replication in them. This may be useful in the development of therapeutics and vaccines of HIV-AIDS [6].

**Figure 1:**
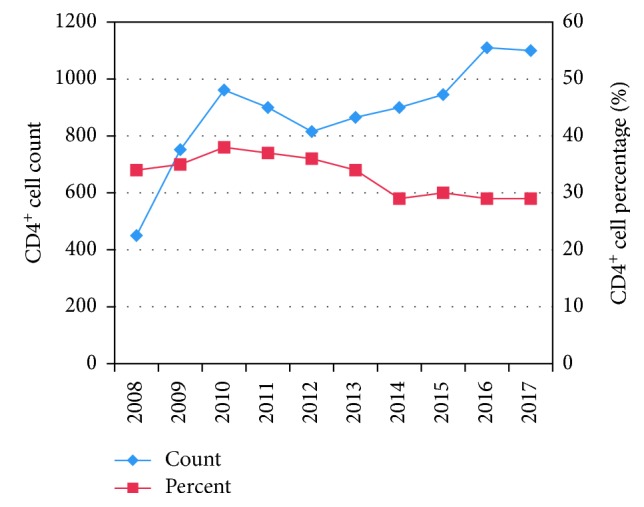
Timeline showing CD4 cell count and its percentage.

In this report, we present a case who had sustained viremic control over HIV infection and maintenance of plasma RNA viral load below 50 copies/ml for more than 10 years. The strong evidence in identifying this case is the laboratory report of viral load and CD4 count with the patient. We found that during the course of 10 years, the patient had constantly maintained the plasma HIV-RNA viral load less than 50 copies/ml and possessed increasing slope of CD4 count (Table 1 and Figure 1). We noted that the patient had decreasing slope of CD4 count in the years 2011 and 2012 though the HIV-RNA viral load was less than 50 copies/ml. We also noted that during such period, the patient had comparatively high percent of CD4 cells. This might be due to some kinds of infection in the patient that have decreased the total WBC count and CD4 count as well.

Several studies have been performed in developed countries regarding elite controllers as well as pathogenesis and host factors but yet have not been able to find any kind of single case from a developing country like Nepal. Probably, this is the first case reporting the evidence of an elite controller in Nepal.

## 4. Conclusions

Detection of elite controllers in HIV-infected patients is a boon for such patients as they could live a normal life without antiretroviral therapy despite HIV infection. On the other hand, this type of cases added further challenges to the researcher in understanding the pathogenesis of HIV as well as other factors responsible for sustained control of viremia in HIV-infected patients. Furthermore, this type of individuals could be used in near future for the development of vaccines and other therapeutics.

## Figures and Tables

**Table 1:** Result of the patient's test report.

Tests	2008	2009	2010	2011	2012	2013	2014	2015	2016	2017
ELISA^∗^	*Positive*	ND	ND	ND	ND	ND	ND	ND	ND	ND
PCR-RNA	ND	<34	TND	TND	<34	TND	TND	TND	<34	<34
Western blot	ND	ND	ND	ND	ND	ND	ND	ND	*Positive*	ND

TND = target not detected; ND = test not done. ^∗^Supplementary testing was done along with ELISA before confirmation.
